# Risk perceptions regarding radiation exposure among Japanese schoolteachers living around the Sendai Nuclear Power Plant after the Fukushima accident

**DOI:** 10.1371/journal.pone.0212917

**Published:** 2019-03-13

**Authors:** Hiroko Hori, Makiko Orita, Yasuyuki Taira, Takashi Kudo, Noboru Takamura

**Affiliations:** 1 Department of Global Health, Medicine and Welfare, Nagasaki University Graduate School of Biomedical Sciences, Nagasaki, Japan; 2 Department of Radioisotope Medicine, Atomic Bomb Disease Institute, Nagasaki University, Nagasaki, Japan; Northwestern University Feinberg School of Medicine, UNITED STATES

## Abstract

In response to the Fukushima Daiichi nuclear power plant disaster, the Nuclear Regulation Authority of Japan issued the new “Nuclear Emergency Response Guideline.” However, there is a perception that scientific information about the health impact of radiation exposure has not been adequately shared among the local government staffs, including schoolteachers. We contacted schoolteachers at all 120 schools within the Urgent Protective Action Planning Zone of the Sendai Nuclear Power Plant, Kagoshima prefecture, in 2017. We invited them to take part in a written survey to clarify their concerns and risk perceptions regarding the effects of radiation exposure on health. Five hundred and fifty schoolteachers’ replies were included in the analysis. The results revealed that 355 schoolteachers had concerns about the health effects of radiation exposure due to working within the Urgent Protective Action Planning Zone. A logistic regression analysis revealed that sex (OR = 2.26, 95% CI: 1.49–3.45, p < 0.001), age (OR = 3.39, 95% CI: 2.10–5.47, p < 0.001), reluctance to undergo a radiological examination at a hospital (OR = 1.91, 95% CI: 1.23–2.88, p = 0.004), place of work (OR = 2.18, 95% CI: 1.46–3.27, p < 0.001), and anxiety about having to address questions about radiation from students (OR = 4.66, 95% CI: 2.83–7.67, p < 0.001) were independently associated with schoolteachers’ concerns about the health effects of radiation exposure due to working in the area around the nuclear power plant. Therefore, it is important to respond to these concerns in order to establish a meaningful education program for school children on radiation and its health effects.

## Introduction

On March 11, 2011, an earthquake led to major problems at the Fukushima Daiichi Nuclear Power Plant (FDNPP). A 14-m tsunami triggered by this earthquake disabled all AC power to Units 1, 2, and 3 of the power plant and carried off fuel tanks for emergency diesel generators. Despite many efforts, cooling systems did not work, and hydrogen explosions damaged the facilities, releasing a large amount of radioactive material into the environment. Although almost 11,000 residents were evacuated from two cities, seven towns, and three villages around the plant during the initial phase of the accident, at present, ten of these have already totally or partially lifted their evacuation orders after exhaustive decontamination efforts and the re-establishment of infrastructure. However, many residents still hesitate to return to their hometowns for various reasons, including employment mismatches, education for their children, and anxiety regarding the health effects of radiation exposure [1, 2]. Although many surveys have shown that the exposure doses of residents are very limited due to the prompt evacuation and food regulation policy, there is a gap between residents’ risk perceptions and their actual exposure doses in Fukushima [1, 3].

On April 26, 1986, the Chernobyl nuclear power plant exploded, emitting tons of radionuclides into the atmosphere and exposing millions of people in Ukraine and neighboring countries to the fallout. Ultimately, 350,000 people living near the plant were permanently relocated, and 600,000 military and civilian personnel from throughout the Soviet Union were recruited as clean-up workers [4]. In addition to clear epidemiological evidence of a rapid increase in childhood thyroid cancers [5], from a public health perspective, the largest impact of the Chernobyl disaster throughout the years has been on mental health, specifically major depression, anxiety disorders, post-traumatic stress disorder (PTSD), stress-related symptoms, and medically unexplained physical symptoms [6]. Given the long-lasting health consequences of natural and man-made disasters, health surveillance and treatment programs are critical in management of health conditions, and emergency preparedness plans, including information sharing with local residents and nuclear emergency training, are needed to prevent or minimize the impact of future threats.

In response to the accident at FDNPP, all of Japan’s nuclear power reactors were stopped. The Nuclear Regulation Authority of Japan issued a new “Nuclear Emergency Response Guideline” in October 2012 for all those nuclear power plants [7]. This new guideline stipulates that appropriate emergency plans should be developed during normal situations to prevent future disasters and to ensure nuclear security. This can enable the application of international criteria, such as Emergency Action Level (EAL) and Operational Intervention Level (OIL), while carrying out preventive protective action and emergency protective action during the initial response stage. In addition, there are now two emergency planning zones (EPZs) defined around each nuclear power plant. The “Precautionary Action Zone (PAZ)” is the area within a radius of 3–5 km, where arrangements should be made to carry out precautionary urgent protective actions for preventing or mitigating the occurrence of severe deterministic effects. Further, the “Urgent Protective Action Planning Zone” refers to the area within a radius of 5–30 km, where preparations should be made to provide prompt shelter, monitor the environment, and implement urgent protective actions based on the results from monitoring the area within a few hours after the release of radionuclides [7]. During a radiological emergency at a nuclear power plant, the overall responsibility of decision-making and taking the appropriate protective actions in the public’s interest rests with the central and local governments [7, 8]. They are also responsible for informing the public about protective measures, such as evacuation, taking shelter in a designated place, or taking potassium iodide supplements [7, 8].

**Fig 1:**
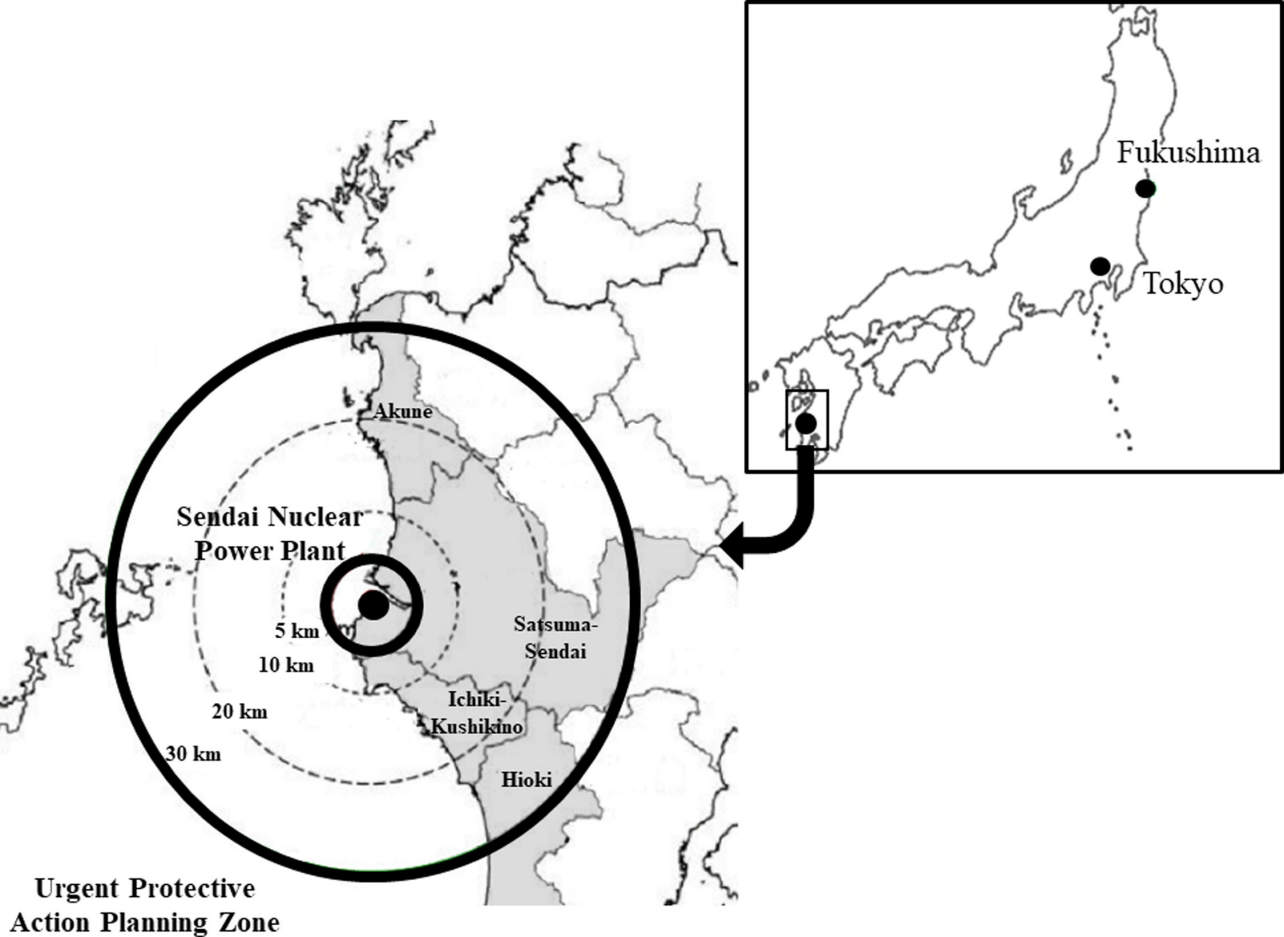
Location of Sendai nuclear power plant in Kagoshima prefecture.

The Sendai Nuclear Power Plant, in southern Japan (Fig 1), was the first to restart operations after the shutdown of all of Japan's nuclear power reactors after the Fukushima accident, in accordance with the new standard. As it was restarted, according to the emergency radiation medical training outlined by the Ministry of Education, Culture, Sports, Science, and Technology [9], local municipalities implemented disaster training with local residents in order to enable people to protect their health from the nuclear emergency by providing information through risk communication. Although nuclear emergency training, including a basic knowledge of radiation and protection from radiation exposure, has also been implemented for all local municipality staff members, including schoolteachers [10], it is assumed that one’s sense of safety regarding radiation exposure and behavioral intentions are associated with one’s risk perceptions. Therefore, risk perception is reported to be associated with sex, age, the presence of children, knowledge, and cultural worldview [11, 12]. Nevertheless, the local municipality staff members’ and schoolteachers’ sense of safety and behavior intentions have not been adequately examined.

Schoolteachers are responsible for teaching their students. Education in Japan includes 6 years of elementary school, 3 years of junior high school, 3 years of high school, and 2 or 4 years of college or university, respectively. Elementary school and junior high school are compulsory. The curriculum studied in schools is prescribed in guidelines called “Courses of Study,” which are established by the MEXT. The school curriculum consists of various academic subjects, moral education, special activities, and integrated studies, which are based on the “Courses of Study.” Public school teachers work for local governments. Elementary school teachers usually have one homeroom class, and they teach their students several subjects. Junior high school teachers usually have one homeroom class, but they teach a specialized subject to several other classes [13]. Because the topic of radiation had been removed from compulsory education in 1977 [14], the general public has not had the opportunity to learn about radiation. However, the curriculum guidelines were revised in 2008, and junior high school students began studying radiation as part of the science program in 2011 [9]. It is assumed that schoolteachers have concerns about sharing correct radiation exposure information and its effects on health with the public and their students because they did not have the opportunity to learn about the health effects of radiation themselves. However, the schoolteachers’ concerns about radiation exposure and its health effects have not been suitably addressed. Accordingly, this study aims to clarify these concerns on the part of schoolteachers regarding the health effects of radiation exposure.

## Methods

### Study participants

We conducted the study in February 2017 in four cities located within the Urgent Protective Action Planning Zone of the Sendai Nuclear Power Plant, Kagoshima prefecture, Japan (Fig 1). Specifically, we distributed a written questionnaire survey to approximated 1,400 teachers who worked in 120 schools (70 elementary schools and 50 junior high schools) located within the Urgent Protective Action Planning Zone of the Sendai Nuclear Power Plant, that is, within 30 km of the nuclear power plant, as established by the Nuclear Regulatory Authority. We obtained responses from 550 teachers (301 males (54.7%) and 249 females (45.3%)). Minors were not included in this study. We confirmed that written consent was obtained from the subjects. The study received approval from the ethics committee of the Nagasaki University Graduate School of Biomedical Sciences (No. 17030211).

### Questionnaire

We developed the written questionnaire for this study based on those used in previous studies to clarify respondents’ concerns and risk perceptions relating to the effects of radiation exposure on health [3, 15–19] (S1 and S2 Figs). It contained questions about the demographic characteristics of the study subjects, including their age, sex, and marital status. Other demographic questions collected information regarding whether there were children in the respondents’ households, whether their houses were over 30 km away from the nuclear plant, the total number of years they had lived at their current residence, the total number of years they had worked as teachers, their current working status and location, and whether they taught science subjects. In addition, we included questions intended to evaluate the schoolteachers’ concerns regarding the health effects of radiation exposure. These addressed whether they were concerned about the potential health effects of radiation because they worked within the Urgent Protective Action Planning Zone, whether they had difficulty conducting classes on radiation, and whether they experienced anxiety about having to address their students’ questions about radiation. We asked the subjects to describe their age, length of residence, and length of teaching career. The other questions were answered by selecting one of the following choices: 1) Yes, 2) Probably, 3) Probably not, and 4) No.

### Statistical analysis

We defined the “concerns (+) group” as “teachers who were concerned about the health effects of radiation exposure from working within the Urgent Protective Action Planning Zone” and the “concerns (−) group” as “teachers who were not concerned about the health effects of radiation exposure from working within the Urgent Protective Action Planning Zone.”

Using the chi-square test, we identified the factors associated with our study subjects’ concerns about the health effects of radiation exposure from working within the Urgent Protective Action Planning Zone. By employing a logistic regression analysis, we calculated the odds ratios (OR) to identify the factors that were independently associated with concerns about the health effects of radiation exposure from living within the Urgent Protective Action Planning Zone. In the analysis, we considered p-values less than 0.05 to be significant. We performed statistical analyses using IBM’s SPSS Statistics 23 software (Armonk, New York).

## Results

Among the 550 study participants, 355 (64.5%) were concerned about the health effects of radiation exposure due to living within the Urgent Protective Action Planning Zone of the Sendai Nuclear Power Plant (“concern (+) group”), whereas 195 (35.5%) schoolteachers were not concerned about this (“concern (−) group”) (S1 Table). Furthermore, 435 schoolteachers (79%) responded that they were anxious about having to address their students’ questions about radiation. In addition, 523 schoolteachers (95%) stated that they had difficulty conducting classes on radiation.

Compared with male teachers, female teachers accounted for a significantly higher percentage of concern (+) respondents than concern (−) respondents (53.2% vs. 30.8%, p < 0.001). The following personal characteristics and health risk perception factors were also significantly associated with a higher proportion of concern (+) as compared to concern (−) responces: participants who were 40 years and older (83.7% vs. 65.6%, p < 0.001), those who had been living in their current residence for more than 7 years (34.6% vs. 24.1%, p < 0.001), those who were concerned about daily life while working within the Urgent Protective Action Planning Zone (95.2% vs. 13.3%, p < 0.001), and those who were reluctant to undergo a radiological examination at a hospital (41.7% vs. 22.1%, p < 0.001) (Table 1).

**Table 1:** Factors associated with schoolteachers’ concerns about the health effects of radiation exposure from working within the Urgent Protective Action Planning Zone (personal characteristics and health risk perceptions).

Unit	Concerns (+) n = 355 (%)	Concerns (-) n = 195 (%)	p-value
**Older than 40 years**	297 (83.7)	128 (65.6)	<0.001
**Female**	189 (53.2)	60 (30.8)	<0.001
**Single household**	80 (22.5)	58 (29.7)	0.062
**Living with children younger than 15 years**	155 (43.7)	82 (42.1)	0.72
**Living within 30 km from the nuclear power plant**	195 (54.9)	97 (49.7)	0.24
**Living in the area for more than 7 years**	123 (34.6)	47 (24.1)	<0.001
**Received training on nuclear emergency preparedness and response**	113 (31.8)	64 (32.8)	0.81
**Listened to a lecture providing basic knowledge on radiation**	68 (19.2)	34 (17.4)	0.62
**Experienced anxiety about daily life while working within the Urgent Protective Action Planning Zone**	338 (95.2)	26 (13.3)	<0.001
**Reluctant to undergo a radiological examination at a hospital**	148 (41.7)	43 (22.1)	<0.001

The numbers refer to the survey participants who responded with a “yes.” The percentages refer to the proportion of participants who responded with a “yes.”

The following factors regarding teaching-related concerns were associated with a significantly a higher proportion of concern (+) as compared to concerns (−) responses: participants who worked at an elementary school (69.9% vs. 49.2%, p < 0.001), those who had worked for more than 21 years (65.1 vs. 45.6%, p < 0.001), those who worked as general teachers (85.6% vs. 82.6%, p < 0.001), those who had experienced being asked about radiation by their student (9.6% vs. 4.1%, p = 0.02), those who could be asked about radiation by their student (62.3% vs. 50.3%, p = 0.006), those who were anxious about having to address their students’ questions about radiation (88.7% vs. 61.5%, p < 0.001), and those who had difficulty conducting classes on radiation (97.2% vs. 91.3%, p = 0.002) (Table 2).

**Table 2:** Factors associated with the schoolteachers’ concerns about the health effects of radiation exposure from working within the Urgent Protective Action Planning Zone (teaching-related concerns).

Unit	Concerns (+) n = 355 (%)	Concerns (-) n = 195 (%)	p-value
**Works at an elementary school, not a junior high school**	248 (69.9)	96 (49.2)	<0.001
**Has worked for more than 21 years**	231 (65.1)	89 (45.6)	<0.001
**Works as a general teacher, not a principal teacher** ^**a**^	304 (85.6)	161 (82.6)	<0.001
**Works as a science teacher**	40 (11.3)	31 (15.9)	0.12
**Experienced being asked about radiation by their students**	34 (9.6)	8 (4.1)	0.02
**Have the possibility to be asked about radiation by their students**	221 (62.3)	98 (50.3)	0.006
**Experienced anxiety about having to address students’ questions about radiation**	315 (88.7)	120 (61.5)	<0.001
**Currently have opportunities to conduct classes on radiation**	109 (30.7)	69 (35.4)	0.26
**Experienced difficulty conducting classes about radiation**	345 (97.2)	178 (91.3)	0.002
**Has referred to books for information on radiation for these classes**	62 (17.5)	37 (19.0)	0.66

The numbers refer to the survey participants who responded with a “yes.” The percentages refer to the proportion of participants who responded with a “yes.”

^a)^ Working status is divided into teachers in charge of a class and principal teachers in charge of managerial work. Principal teachers are selected from the general teachers.

The logistic regression analysis revealed that sex (OR = 2.26, 95% CI: 1.49–3.45, p < 0.001), age (OR = 3.39, 95% CI: 2.10–5.47, p < 0.001), reluctance to undergo a radiological examination at a hospital (OR = 1.91, 95% CI: 1.23–2.88, p = 0.004), place of work (OR = 2.18, 95% CI: 1.46–3.27, p < 0.001), and anxiety about having to address their students’ questions about radiation (OR = 4.66, 95% CI: 2.83–7.67, p < 0.001) were independently associated with the schoolteachers’ concerns regarding the health effects of radiation exposure from working within the Urgent Protective Action Planning Zone (Table 3).

**Table 3:** Logistic regression analysis of schoolteachers’ concerns about the health effects of radiation exposure from working within the Urgent Protective Action Planning Zone.

Variables	Unit	OR^a^	95% CI^b^	p-value
**Sex**	Female/Male	2.26	1.49–3.45	<0.001
**Age**	40 years or older/20–39 years old	3.39	2.10–5.47	<0.001
**Number of years of residing in the area**	Less than 7 years/More than 7 years	0.82	0.52–1.30	0.40
**Place of work**	Elementary school/Junior high school	2.18	1.46–3.27	<0.001
**Anxious about having to address students’ questions about radiation**	Yes/No	4.66	2.83–7.67	<0.001
**Reluctant to undergo a radiological examination at a hospital**	Yes/No	1.91	1.23–2.98	0.004

^a^ OR: Odds Ratio

^b^ 95% CI: 95% confidence interval

## Discussion

In this study, we identified the concerns of schoolteachers regarding the health effects of radiation exposure due to working in the Urgent Protective Action Planning Zone around the Sendai Nuclear Power Plant. The findings revealed that 64.5% of the teachers expressed these concerns and that sex, age, reluctance to undergo a radiological examination at a hospital, place of work, and anxiety about having to address their students’ questions about radiation were independently associated with the schoolteachers’ concerns regarding the health effects of radiation exposure due to working within the Urgent Protective Action Planning Zone. In a previous study conducted in 2013, we administered a survey to gauge risk perception among the clinical nurses working at Fukushima Medical University Hospital, Fukushima Prefecture; the survey results showed that 71.5% of the nurses expressed anxiety about radiation exposure [15]. The Japanese government, research institutes, [2, 20–22] and international authorities, such as the International Atomic Energy Agency (IAEA) [23], the World Health Organization (WHO) [24], and the United Nations Scientific Committee on the Effects of Atomic Radiation (UNSCEAR) [25], reported that there would be no detectable direct health effects of radiation exposure on the general public following the accident at the Fukushima Daiichi nuclear power plant. Despite this, residents of Japan—even those in professional occupations, such as schoolteachers—continue to have concerns about the radiological effects. These concerns have probably stemmed from the lack of knowledge about radiation exposure and its health effects, which even exists among educated, individuals in professional occupations.

The logistic regression analysis showed that several personal characteristics and health risk perception factors, including sex, age, and reluctance to undergo a radiological examination at a hospital, were independently associated with teachers’ concerns about the health effects of radiation exposure due to working within the Urgent Protective Action Planning Zone of the Sendai Nuclear Power Plant. Consistent with previous studies, the results of our survey indicated that female participants expressed greater concerns about the health effects of radiation exposure as compared to their male counterparts [8, 19]. In addition, we found that schoolteachers older than 40 years expressed greater concerns about the health effects of radiation exposure as compared to their younger counterparts. However, our earlier study from 2013 showed that the intention to quit work was significantly associated with younger nurses and nurses with shorter tenures [15]. These nurses were being studied in association with a nuclear plant disaster, and the survey linked the intent to leave their job with the stress of the nuclear disaster. In another previous study, we surveyed non-medical employees who had been working in the surrounding areas of the Fukushima Daiichi nuclear power plant when the accident took place there; the objective was to determine the factors associated with their intention to quit during the nuclear disaster. The survey revealed that age and employee tenure was not significantly different between employees who intended to quit and those who had no intention to leave their jobs [16]. Finally, Murakami et al. stated that anxiety about radionuclides can be explained by characteristics of human perception concerning the risks of radiation and a distrust of the government and experts [17]. Such inconsistent results may be due to occupational differences, the influence of a sense of responsibility at work following a nuclear disaster, and the personal characteristics of individuals that guide how they understand the radiological risks.

In addition, our results showed that place of work and anxiety about having to address their students’ questions about radiation were independently associated with schoolteachers’ concerns regarding the health effects of radiation exposure due to working within the Urgent Protective Action Planning Zone. The results indicated that teachers who worked at elementary schools and those concerned about having to address their students’ questions about radiation experienced greater anxiety relating to radiation exposure and its health effects. One potential reason for this is stress relating to teaching students and school children about radiation and its health effects. Because it is absolutely essential to provide students and school children with comprehensive, ongoing education about the risks of radiation [26], schools play an important role in the dissemination of accurate information about radiation and risk awareness [27–29]. On the other hand, our present results showed that the questionnaire items “received questions from students about radiation,” “opportunities to conduct classes on radiation,” and “referred to books for information on radiation” received relatively few responses. Because, in the Japanese education system, the topic of radiation was removed from compulsory education in 1977 [14], the general population—especially an entire generation of young and middle-aged adults—has not had the opportunity to learn about radiation. Such limited opportunities may pose challenges, even for schoolteachers, in obtaining accurate information about the risks of radiation.

Nevertheless, the curriculum guidelines were revised in 2008 [9]. After the Fukushima Daiichi nuclear power plant accident, junior high school students began studying radiation as part of the science programs since 2012. In addition, Japan’s Ministry of Education, Culture, Sports, Science and Technology is preparing a new education program for medical students in universities, which will cover the risks of radiation for human health [30]. Although there has been no decision to implement compulsory education on radiation in elementary schools, the number of students being taught this subject from a young age has been gradually increasing across Japan.

Another issue is that teachers may face anxiety if they must follow a radiation curriculum and teach classes that are entirely new. Because teachers, especially those teaching in elementary schools, lack opportunities to obtain accurate information about the risks of radiation, they may perceive radiological risks as unknown risks, which may result in anxiety about radiation exposure and its health effects. This suggests the importance of developing a comprehensive education program for teachers to improve the educational environment and alleviate their concerns about radiation exposure and its health effects via collaboration between teachers, experts, and local governments.

The present study has several limitations. First, there might be a participant bias since this study was conducted only in the Urgent Protective Action Planning Zone of the Sendai nuclear power plant. This also limits the generalizability of the findings. Through the questionnaire, we were unable to obtain sufficient information on potential confounding factors, such as detailed lifestyle habits, the educational environment, and the teachers’ sense of responsibility in performing their duties as educators with respect to nuclear disaster.

In conclusion, this study revealed that schoolteachers’ sex, age, reluctance to undergo a radiological examination at a hospital, place of work, and anxiety about having to address their students’ questions about radiation were independently associated with their concerns about the health effects of radiation exposure from working within the Urgent Protective Action Planning Zone of the nuclear power plant. Therefore, it is important to respond to these concerns of schoolteachers in order to establish a meaningful education program for school children on radiation and its health effects.

## Supporting information

S1 Fig Original question sheet in Japanese. (DOC)

S2 Fig Translation question sheet. (DOC)

S1 Table Analysis data sheet. (XLSX)
