# Supplementary material for: Risk perceptions regarding radiation exposure among Japanese schoolteachers living around the Sendai Nuclear Power Plant after the Fukushima accident
Source: PLoS One. 2019 Mar 13;14(3):e0212917. doi: 10.1371/journal.pone.0212917 (PMC6415797; doi:10.1371/journal.pone.0212917)
Supplement: S1 Fig — (DOC) [file pone.0212917.s001.doc]

**原子力発電所UPZ内の自治体職員と教職員における放射線リスク認知調査**

以下の質問への回答にご協力下さい。空欄への語句や数字の記入、または、最も該当する番号に○をおつけ下さい。

質問1　年齢を教えて下さい。　　　( )歳

質問2　性別を教えて下さい。　　　1) 男性 2) 女性

質問3　何人暮らしですか。　　　　1) 単身　　　　　　　 2) 二人以上

質問4　家族に15歳以下のお子さんがいらっしゃいますか。　1) いる　　　2) いない

質問5　どちらにお住まいですか？　川内原子力発電所から概ね、

1)　5km内　　2) 10km内　　3) 20km内　　4) 30km内　　5) 30km外　　6) わからない

質問6　現在のお住まいでの居住年数を教えて下さい。(　　　　　)年

質問7　職場はどちらですか。　　　1）小学校　 　　　　　2）中学校

質問8　勤続年数を教えて下さい。(　　　　　)年

質問9　現在の職位に最も当てはまるものをお選び下さい。　　1）教諭　　2）教頭以上

質問10　あなたは理科系教諭ですか。1）はい　　　　　　　2）いいえ

質問11　これまで原子力防災訓練に参加されたことがありますか？　　1) はい　　2) いいえ

質問12　これまで放射線についての研修に参加されたことがありますか。　1) はい　　2) いいえ

質問13　現在、原子力発電所UPZ区域内で勤務していることで生活全般に対して不安がありますか。

1)　不安を感じる　　　　　　2)　やや不安を感じる

3)　あまり不安を感じない　　4)　不安を感じない

質問14　現在、原子力発電所UPZ区域内で勤務していることで、放射線に対して不安がありますか。

1)　不安を感じる　　　　　　2)　やや不安を感じる

3)　あまり不安を感じない　　4)　不安を感じない

質問15　医療機関で、胸部レントゲン、CT、心臓カテーテル、マンモグラフィーなどの放射線を利用した検査を受けることに抵抗がありますか？

1) ある　　　　　2) ない

質問16　これまでに職務中で、児童・生徒から放射線関連（原子力関連含む）の相談・質問を受けたことがありますか。

1) ある　　　　2) ない

質問17　今後、職務中に、児童・生徒から、放射線関連（原子力関連含む）の相談・質問を受ける可能性はありますか。

1) ある　　　　2) ない

質問18　児童・生徒からの放射線関連（原子力関連含む）の相談・質問を受けることに不安を感じますか。

1)　不安を感じる　 　2)　やや不安を感じる

3)　あまり不安を感じない　　4)　不安を感じない

質問19　現在のあなたの職務内容では、放射線関連(原子力防災を含む)の授業を生徒に行う機会がありますか？

1) ある　　　　2) ない

質問20　現在、あなたが放射線関連(原子力防災を含む)の授業を行うとしたら困難感を感じますか？

1)　とても困難感を感じる　 2)　少し困難感を感じる

3) あまり困難感を感じない　3) 全く困難感を感じない

質問21　生徒に放射線教育(原子力防災を含む)を行う時、「放射線副読本」を活用されていますか？

1) とても活用している　　　2) 少し活用している

3) あまり活用していない　　4) 全く活用していない

ご協力ありがとうございました。以上でアンケートは終わりです。
