# Supplementary material for: Risk perceptions regarding radiation exposure among Japanese schoolteachers living around the Sendai Nuclear Power Plant after the Fukushima accident
Source: PLoS One. 2019 Mar 13;14(3):e0212917. doi: 10.1371/journal.pone.0212917 (PMC6415797; doi:10.1371/journal.pone.0212917)
Supplement: S2 Fig — (DOC) [file pone.0212917.s002.doc]

**Questionnaire**

Please answer the following questions and circle that apply.

1. Age:　 (　　　　　)years old

2. Sex: 1) Male 2) Female

3. How many people are you living in?

1) Single household 2) 2 people or more

4. Are you living with children younger than 15 years?

1) Yes 　　 2) No

5. Are you living within 30 km from the nuclear power plant?

1)　Yes　　2) No　　3) I do not know

6. How many years are you living in this area?

(　　　 )years

7. Where do you work in?

1）Elementary School 　 2）Junior High School

8. How long have you been working as a school teacher?

( )years

9. What is your current position?

1）General teacher　　2）Principal teacher

10. Are you a science teacher? 1）Yes　　　　　　　2）No

11. Have you ever received training on nuclear emergency preparedness and response?

1) Yes 2) No

12. Have you ever participated in a lecture on radiation providing basic knowledge?

1) Yes 2) No

13. Have you ever experienced anxiety about daily life while working within the Urgent Protective Action Planning Zone?

1)　Yes　　　　　　2)　Probably 3)　Probably no　　4)　No

14. Do you have concerns about the health effects of radiation exposure from living within the Urgent Protective Action Planning Zone?

1)　Yes　　　　　　2)　Probably 3)　Probably no　　4)　No

15. Are you reluctant to undergo a radiological examination at a hospital?

1) Yes 2) No

16. Have you ever received questions about radiation from students?

1) Yes 2) No

17. May you receive questions about radiation from students?

1) Yes 2) No

18. Have you ever experienced anxiety about having to address students’ questions about radiation?

1)　Yes　　　　　　2)　Probably 3)　Probably no　　4)　No

19. Currently, do you have opportunities to conduct classes on radiation?

1) Yes 2) No

20. Have you experienced feeling difficulty in conducting classes about radiation about radiation?

1)　Yes　　　　　　2)　Probably 3)　Probably no　　4)　No

21. Have you ever referred to books for information on radiation for these classes?

1)　Yes　　　　　　2)　Probably 3)　Probably no　　4)　No

Thank you for your cooperation.
